# Clinical and cost-effectiveness of progressive exercise compared with best practice advice, with or without corticosteroid injection, for the treatment of rotator cuff disorders: protocol for a 2x2 factorial randomised controlled trial (the GRASP trial)

**DOI:** 10.1136/bmjopen-2017-018004

**Published:** 2017-07-17

**Authors:** Sally Hopewell, David J Keene, Michael Maia Schlüssel, Melina Dritsaki, Susan Dutton, Andrew Carr, William Hamilton, Zara Hansen, Anju Jaggi, Chris Littlewood, Hessam Soutakbar, Peter Heine, Lucy Cureton, Karen Barker, Sarah E Lamb

**Affiliations:** 1 Nuffield Department of Orthopaedics, Rheumatology and Musculoskeletal Sciences, University of Oxford, Oxford, UK; 2 Medical School, University of Exeter, Exeter, UK; 3 Royal National Orthopaedic Hospital NHS Trust, Stanmore, Middlesex, UK; 4 Primary Care and Health Sciences, Keele University, Keele, UK; 5 Oxford University Hospitals NHS Trust, Oxford, UK

**Keywords:** rehabilitation medicine, primary care, pain management, musculoskeletal disorders

## Abstract

**Introduction:**

Shoulder pain is very common, with around 70% of cases due to disorders of the rotator cuff. Despite widespread provision of physiotherapy, there is uncertainty about which type of exercise and delivery mechanisms are associated with best outcomes. There is also uncertainty around the long-term benefits and harms of corticosteroid injection therapy, which is often used in addition to physiotherapy. The Getting it Right: Addressing Shoulder Pain trial will assess the clinical and cost-effectiveness of individually tailored, progressive exercise compared with best practice advice, with or without corticosteroid injection, in adults with a rotator cuff disorder.

**Methods and analysis:**

We are conducting a large multicentre randomised controlled trial (2×2 factorial design). We will recruit adults ≥18 years with a new episode of shoulder pain attributable to a rotator cuff disorder as per British Elbow and Shoulder Society guidelines, not currently receiving physiotherapy or being considered for surgery, from at least eight UK National Health Service primary care-based musculoskeletal and related physiotherapy services. Participants (n=704) will be randomised (centralised computer-generated 1:1:1:1 allocation ratio) to one of four interventions: (1) progressive exercise (≤6 physiotherapy sessions); (2) best practice advice (one physiotherapy session); (3) corticosteroid injection then progressive exercise (≤6 sessions) or (4) corticosteroid injection then best practice advice (one session). The primary outcome is the mean difference in Shoulder Pain and Disability Index (SPADI) total score at 12 months. Secondary outcomes are: pain and function SPADI subdomains; health-related quality of life (Five-Level version of the EuroQol EQ-5D-5L); sleep disturbance; return to activity; global impression of change; health resource use; out-of-pocket expenses; work disability. A parallel within-trial economic evaluation will be conducted. The primary analysis will be intention to treat.

**Ethics and dissemination:**

Research Ethics Committee approval (REC: 16/SC/0508) has been obtained. Results of the main trial and secondary outcomes will be submitted for publication in a peer-reviewed journal.

**Trial registration number:**

ISRCTN16539266; EudraCT number: 2016-002991-28.

**Strengths and limitations of this study**

- The Getting it Right: Addressing Shoulder Pain trial is a large multicentre randomised controlled trial based in primary care and primary care interface services.
- Using a factorial design, we will cotest two interventions commonly used together in the management of rotator cuff disorders in primary care: progressive exercise delivered by a physiotherapist and corticosteroid injection. We will use a best-practice advice session with a physiotherapist and no injection as the comparators.
- We want to assess which of these interventions, or combination of interventions, are most clinically and cost-effective. A parallel within-trial health economic analysis will also be conducted for the period of the trial.
- All outcome measures are patient reported. Due to the nature of the interventions, it is not possible to blind the study participants or care providers.

## Introduction

Shoulder pain is common. The prevalence of shoulder complaints in the UK is estimated at around 14%,^1^ increasing with age^2^ and highest in those aged 60 and above. Annually, around 1% of adults aged over 45 in primary care present with a new episode of shoulder pain,^3^ the most common attribution being the rotator cuff which accounts for around 70% of cases.^3^ Disorders of the rotator cuff are associated with substantial disability and pain and can have a significant impact on patient health and well-being, affecting an individual’s capacity to work and ability to perform daily tasks and social activities. Problems can persist for long periods with up to half of those who present for care continuing to have pain and/or functional disturbance for up to 2 years.^4^

The majority of shoulder pain is managed in primary care or at primary care interface musculoskeletal services by physiotherapists and general practitioners (GPs). The aim of treatment is to improve pain and function, and standard primary care options include rest, advice, analgesia, non-steroidal anti-inflammatory drugs, referral to physiotherapy and corticosteroid injections.^5 6^ However, usual care can be highly variable. Furthermore, there are no recommended National Institute for Health and Care Excellence clinical guidelines for this area.

There is promising evidence from small, short-term trials^7 8^ that physiotherapist-prescribed exercise is effective for the treatment of rotator cuff disorders. However, a recent Cochrane review^9^ highlighted the lack of evidence about its long-term clinical and cost-effectiveness, despite the widespread provision of physiotherapy for these conditions. There is also uncertainty about which types of exercise and delivery mechanisms are associated with the best outcomes.^7 8 10–12^ This evidence is limited by problems in study design and choice of comparators.^8^ There are also competing ideologies around which type of exercise programmes are considered most important. Resistance training to improve muscular strength, whether supervised or home based, has been identified as a core component of exercise for rotator cuff disorders, although there is no evidence that any specific programme is superior.^13 14^ In a trial of strength training, duration, specificity of exercises, progression criteria and individualisation (ie, adjusting the programme to suit each participant) were also considered important.^15^ In addition, little attention has been paid to the need for behavioural frameworks to enhance adherence to exercise, which is predominantly performed without physiotherapist supervision, and tackle unhelpful pain beliefs and behaviour.^16^

Corticosteroid injections are commonly used to reduce pain and inflammation associated with rotator cuff disorders. There is good systematic review evidence^17–19^ that, in comparison with placebo, corticosteroid injections have a short-term benefit in the shoulder, as in other areas of the body. However, there are some concerns about their longer-term safety.^20^ The combination of injection and physiotherapy has intuitive appeal, with some evidence of an additive, but not interactive, short-term effect.^19 21–23^ Due to the longer-term safety concerns, we believe that they require more study and hence have included unguided corticosteroid injections as part of our study design. Although the use of ultrasound to guide injections in primary care has become increasingly common, emerging evidence from the SUbacromial imPingement syndrome and Pain: a randomised cOntrolled Trial of exercise and injection (SUPPORT) trial (which looked at guided vs unguided injection) and others have demonstrated that it is no more effective than standard injection practice.^4 24^

The UK National Health Service (NHS) currently invests considerable resources on unproven therapies and must develop cost-effective, pragmatic methods of dealing with high-volume conditions such as rotator cuff disorders. The consequences of poor initial management can lead to an increased likelihood of recurrent or persistent problems in older age and the need for surgery.^5^ Based on the existing evidence, we propose a definitive randomised controlled trial, using a factorial design, to cotest two interventions commonly used together in the management of rotator cuff disorders in primary care: progressive exercise delivered by a physiotherapist and corticosteroid injection. We will use a best-practice advice session with a physiotherapist and no injection as the comparators. We want to assess which of these interventions, or combination of interventions, are most clinically and cost-effective.

## Objectives

The primary objectives of the Getting it Right: Addressing Shoulder Pain (GRASP) trial are to assess whether (1) an individually tailored progressive home exercise programme prescribed and supervised by a physiotherapist provides greater improvement in shoulder pain and function at 12 months compared with a best practice advice session with a physiotherapist supported by high-quality self-management materials and whether (2) subacromial corticosteroid injection provides greater improvement in shoulder pain and function at 12 months compared with no injection.

Secondary objectives are to investigate if there are any differences at 8 weeks, 6 and 12 months in the following: shoulder pain; shoulder function; health-related quality of life; psychological factors; sleep disturbance; return to desired activities including work, social life and sport activities; patient global impression of change; adherence to exercises, use of medication (prescribed and over the counter); time off work; health resource use (consultation with primary and secondary care) and additional out-of-pocket expenses. A parallel within-trial health economic analysis will also be conducted for the period of the trial.

## Methods and analysis

### Study design

**Figure 1:**
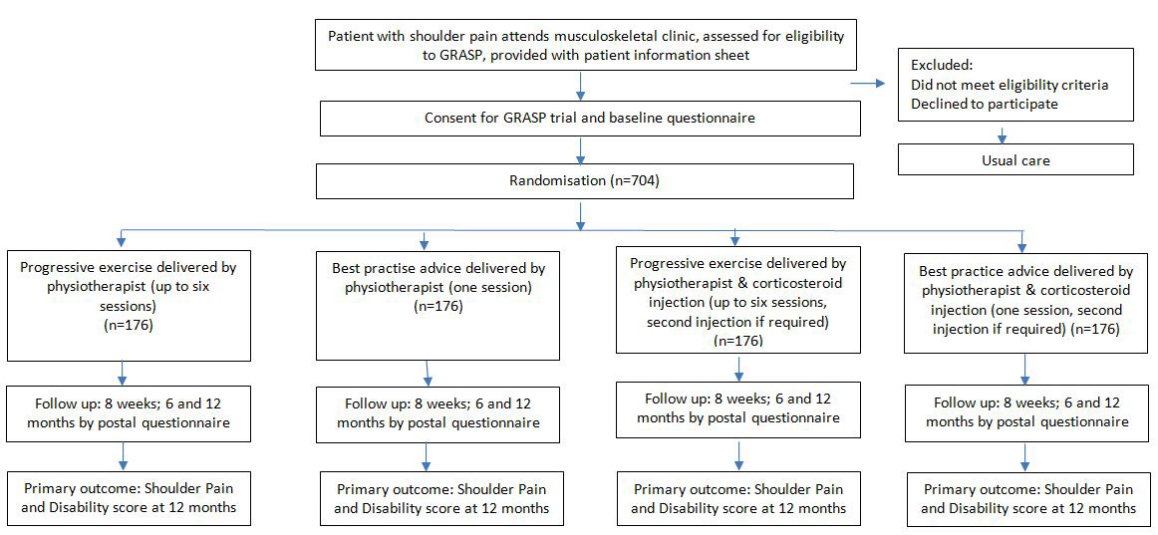
Study flow diagram for GRASP trial. GRASP, Getting it Right: Addressing Shoulder Pain.

The study will be a 2×2 factorial randomised controlled trial design (figure 1).

### Setting

The trial will be conducted across at least eight primary care-based musculoskeletal services and their related physiotherapy services in the UK NHS. These services treat people with a range of musculoskeletal conditions and are run by specialist practitioners including extended-scope physiotherapists and GPs with a specialist interest in musculoskeletal conditions. Sites will be chosen so they reflect a range of settings (urban and rural) and are able to deliver the trial interventions.

### Study participants

Participants will be recruited if referred by their GP or physiotherapist for treatment of a new episode, but not necessarily first episode of shoulder pain attributable to a rotator cuff disorder. Participants should be predominantly seeking treatment for one shoulder. People who self-refer directly to the service will also be assessed for eligibility as the typical route of referral can vary across services. The participants will not undergo diagnostic imaging such as MRI or ultrasound as a requirement of the trial, as this is generally not recommended in primary care.^5^

#### Eligibility

Patients will be eligible for this study if they are:

- aged 18 years and above;
- have a new episode of shoulder pain (ie, within the last 6 months) attributable to a rotator cuff disorder (eg, cuff tendonitis, impingement syndrome, tendinopathy or rotator cuff tear) using the diagnostic criteria set out in the British Elbow and Shoulder Society (BESS) guidelines^5^ (online supplementary appendix 1);
- not currently receiving physiotherapy;
- not being considered for surgery;
- able to understand spoken and written English.

10.1136/bmjopen-2017-018004.supp1 Supplementary Appendix 1

Patients will be excluded from participation in this study if:

- there is a history of significant shoulder trauma (eg, dislocation, fracture or full thickness tear requiring surgery);
- there is a neurological disease affecting the shoulder;
- they have other shoulder disorders (eg, inflammatory arthritis, frozen shoulder, glenohumeral joint instability) or have red flags consistent with the criteria set out in the BESS guidelines^5^;
- they have received corticosteroid injection or physiotherapy for shoulder pain in the last 6 months;
- they have contraindications to corticosteroid injection.

### Recruitment of participants, screening and eligibility assessment

Potential participants will attend their appointment in accordance with standard NHS procedures. The treating practitioner will undertake a clinical assessment according to their usual practice. If a patient fulfils the criteria for a rotator cuff disorder, they will then be assessed to see whether they meet the GRASP eligibility criteria. Patients will be provided with a copy of the participant information sheet and asked if they wish to be considered for the trial. Those meeting the eligibility criteria and wishing to participate in the trial will then be approached for informed consent. Participants who do not meet the eligibility criteria, or who do not wish to participate, will receive standard NHS treatment. We will record anonymous information on the age and sex of those who decline to participate so that we can assess the generalisability of those recruited. The reasons for declining will also be recorded.

### Informed consent and baseline assessment

After participants have been assessed for eligibility, informed consent for participation in the trial will be sought by a research facilitator trained in good clinical practice. Consent may take place during the initial appointment or may require a second appointment if participants require more time to consider the study. Participants will then be asked to complete the baseline assessment questionnaire that will record simple demographic information and baseline measurements for the primary and secondary outcomes (tables 1 and 2).

**Table 1:** Time-points at which the outcomes will be assessed

Outcome	Measurement	Time-point
Demographic	Age, sex, height, weight, ethnicity, marital status, smoking, date of rotator cuff diagnosis, duration of symptoms, hand dominance, affected shoulder, current work status, level of education, place of residence, household income, state benefits	0
*Primary*		
Pain and function	SPADI^37 38^ 13-item total scale	0, 8 weeks, 6 months, 12 months
*Secondary*		
Pain	SPADI^37 38^ five-item subscale	0, 8 weeks, 6 months, 12 months
Function	SPADI^37 38^ eight-item subscale	0, 8 weeks, 6 months, 12 months
Health-related quality of life	EQ-5D-5L score^41^	0, 8 weeks, 6 months, 12 months
Psychological factors	Fear Avoidance Belief Questionnaire physical activity five-item subscale^42^ Pain Self-efficacy questionnaire (short form)^43^	0, 8 weeks, 6 months, 12 months
Sleep disturbance	Insomnia Severity Index^44^	0, 8 weeks, 6 months, 12 months
Global impression of treatment	Patient-rated Likert scale^45^	8 weeks, 6 months, 12 months
Return to desired activities	Patient-reported return to desired activities, including work, social life and sport activities	8 weeks, 6 months, 12 months
Exercise adherence	Patient-reported adherence to exercise	8 weeks, 6 months, 12 months
Medication usage	Prescribed and over-the-counter medications, additional steroid injection	8 weeks, 6 months, 12 months
Work disability	Sick leave (days)	8 weeks, 6 months, 12 months
Healthcare use	NHS outpatient and community services (eg, GP, additional physical therapy) NHS inpatient and day case (eg, radiography, MRI) Private healthcare services	8 weeks, 6 months, 12 months
Out-of-pocket expenses	Patient-related out-of-pocket expenses recording form	8 weeks, 6 months, 12months

EQ-5D-5L, Five-Level version of EuroQol Five Dimensions Questionnaire; GP, general practitioner; NHS, National Health Service; SPADI, Shoulder Pain and Disability Index.

**Table 2:** Participant timeline

Time-point	Pre randomisation	Baseline	0–4 months	8-week follow-up	6-month follow-up	12-month follow-up
Enrolment						
Screening log	✓					
Eligibility confirmed	✓					
Informed consent	✓					
Randomisation		✓				
Interventions						
Steroid injection (if randomised to)			✓			
Progressive exercise intervention (if randomised to)			✓			
Best practice advice intervention (if randomised to)			✓			
Assessments						
Baseline questionnaire	✓					
Follow-up questionnaire				✓	✓	✓
Follow-up reminders				✓	✓	✓

### Randomisation

Consented participants will be randomised to intervention groups (1:1:1:1) by the site research facilitator using the centralised computer randomisation service provided by the Oxford Clinical Trials Research Unit. Randomisation will be computer-generated and stratified by centre, age and gender, using a variable block size to ensure the participants from each site have an equal chance of receiving each intervention. Participants will be randomised to one of four physiotherapy-led interventions:

- Progressive exercise programme: an individually tailored progressive home exercise programme prescribed and supervised by a physiotherapist involving up to six face-to-face sessions over 16 weeks.
- Best practice advice: one face-to-face session with a physiotherapist and a simpler home exercise programme supported by high quality self-management materials.
- Progressive exercise programme (as described above), preceded by a corticosteroid injection.
- Best practice advice session (as described above), preceded by a corticosteroid injection.

### Blinding

Physiotherapists delivering the intervention and study participants will be informed of their treatment by the site research facilitator at the initial appointment and so will not be blinded to the treatment allocation. The trial statistician and data entry personnel will also not be blinded to the treatment allocation. The remaining members of the central trial management team will be blinded to treatment allocation until after the data analysis is complete.

### Interventions

#### Subacromial corticosteroid injection

The subacromial corticosteroid injection will be delivered prior to the progressive exercise or best practice advice intervention, predominately by extended-scope physiotherapists with appropriate postregistration qualifications in injection therapy working within a local patient group directive or equivalent. This reflects an increasingly common practice in the NHS and ensures that the injections are delivered in the most cost-effective manner possible. The corticosteroid injection will be given as per its marketing authorisation and in accordance with its normal indication and therapeutic dosage (www.medicines.org.uk/emc/). The corticosteroid will be given together with local anaesthetic in one injection at the same time, or separately, depending on local treatment protocols at sites.

The corticosteroid injected will be either methylprednisolone acetate (up to 40 mg) or triamcinolone acetonide (up to 40 mg), depending on local treatment protocols at each site. These are the two routinely injected corticosteroids for shoulder pain; there is no clear evidence that either corticosteroid is more effective than another.^19^ The local anaesthetic will be either 1% lidocaine (up to 5 mL) or 0.5% bupivacaine hydrochloride (up to 10 mL), depending on local treatment protocols. We will only select sites that adhere to these prescribing boundaries. The choice and dose of corticosteroid and local anaesthetic (including volume) will be recorded for each participant in the trial data collection forms.

Participants will be advised to take care and avoid heavy lifting for 24–48 hours postinjection. Appointments will be coordinated so that participants typically receive their injection within 10 days of randomisation. Very occasionally, a second injection may be given after 6 weeks (but within 16 weeks of the patient being randomised), but will only be administered to those patients who receive good initial benefit from their first injection and who request further pain relief to facilitate their exercises. Any participants that receive a second injection will have the dose, drug and date of administration recorded in their trial data collection form.

#### Progressive exercise intervention

Participants randomised to the progressive exercise programme will receive up to six sessions with a physiotherapist over 16 weeks. These sessions will have a strong behavioural component to encourage adherence to the home exercise plan. A similar rationale has been used to good effect in other trials.^15 25^ This number of sessions, spread over this time, allows progression of the intensity of exercise and sufficient time for a physiological response in the neuromuscular system to improve function. It also allows time to instigate longer term health behaviour change. Appointments will be coordinated so that participants typically start their first exercise session within 14 to 28 days of randomisation, as per local appointment availability. The initial session will last up to 60 min for assessment and starting the exercise programme, followed by up to five follow-up sessions of 20 to 30 min each. The physiotherapists will record the number of prescribed treatment sessions attended by each participant. The intervention has been designed to ensure that sufficient dose is delivered and to maximise compliance. Importantly, the intervention can be delivered within the current NHS commissioning paradigm.^26^ The progressive exercise programme consists of three phases of progression:

##### Phase 1—Assessment and advice

Participants will be assessed and then given a self-management leaflet and tailored education, reassurance and advice on pain management and activity modification. They will also be given shoulder exercises to practise at home until their next session.

##### Phase 2—Progressive structured resistance training

Resistance exercises will be added that are highly structured and aim to improve the shoulder’s functional capacity. The exercises will be rehearsed in the physiotherapy department and then practised at home. The progression of the volume and load of the resistance training will be based on existing guidelines^27^ and will take into account each individual’s capabilities and preferences. The modified Borg scale of perceived exertion will be used to regulate the intensity of the resistance exercise.^28^ The load will initially be set at a moderate level to permit progression, enhance motivation and adherence and reduce the possibility of symptom flare-up. The exercises will target the patient’s shoulder movement difficulties in a progressive way, consistent with expert consensus.^29^ Progression will be achieved by increasing the resistance/and or the number of repetitions. Hand weights or resistance bands will be used to add resistance. Participants will be advised that some pain during the exercises is acceptable, provided the participant is happy and the symptoms resolve to an acceptable level after exercise.^30^ Patient preference for how each exercise is performed (where, when and position) will be agreed with the physiotherapist. The participants and therapists will negotiate an effective dose of exercise, progressively giving the participant overall control.

##### Phase 3—Patient-specific functional restoration

The final stage of training involves modifying the core resistance training exercises towards the specific strengthening movements required to achieve the functional goals of the individual.

Established behavioural change strategies^31^ will be used to maximise adherence to the exercise intervention. Implementation intentions and action planning techniques^32^ have been found to be effective in improving physical activity levels. These intentions will form part of a behavioural exercise plan and exercise diary, which patients have reported to be helpful in promoting adherence.^33^ The physiotherapists delivering the intervention will be trained in questioning techniques^34^ to elicit and address unhelpful beliefs about shoulder pain or exercise that may impede adherence.^35^

#### Best practice advice session

The participants randomised to the best practice advice session will receive a single face-to-face session with a physiotherapist, lasting up to 60 min. Appointments will be coordinated so that participants typically start their exercise session within 14 to 28 days of randomisation, as per local appointment availability. The best practice advice session will have substantially greater reliance on self-management. After a comprehensive shoulder assessment, the participants will be given a self-management advice leaflet, tailored education, reassurance and self-management exercise advice, including advice on pain management and activity modification. They will also be given a simple set of self-guided exercises that can be progressed and regressed independently depending on their capability. The exercises will be designed using similar concepts to the progressive exercise intervention, such as increased resistance but there will be a simpler range of exercise options and will not be supervised. An exercise diary will be issued and a simplified exercise planner will be completed to encourage adherence with exercise. The best practice advice intervention will not be underpinned by the additional reassurance of follow-up physiotherapy appointments, which also enable more comprehensive behavioural aspects of the intervention through ongoing feedback and assistance with developing problem-solving over time.

The best practice advice session will be supported by high-quality patient self-management information and exercise videos available through a website and digital video disc. As possible low health literacy was a major consideration when developing materials, plain English and patient representative involvement was used to optimise material accessibility. Using different media aims to make the information accessible and more appealing to a wide range of individuals^36^ and has been used successfully in other trials of painful musculoskeletal disorders.

A best practice advice session has been selected as the comparator because it is consistent with current clinical practice guidelines.^5 6^ This intervention also minimises the use of some physiotherapy treatments that, while commonly used, have evidence of no or minimal effect. In addition, many people find a single advice session and videos more beneficial as they do not have to come back to the hospital, take time off work or make carer arrangements. This intervention may best serve commissioners, patients and clinicians in the long term.

#### Concomitant care

All participants will be advised to take over-the-counter analgesia as required, in accordance with the BESS guidelines.^5^ In addition, participants will be provided with advice on modifying activities that exacerbate symptoms and on sleeping positions. Participants may seek other forms of treatment during the trial but will be informed they should use usual routes (predominantly NHS referral) to do so. Additional treatments, including contact with their GP or other health professional, changes in medication, use of physical treatment and alternative therapies, will be recorded as a treatment outcome.

### Training and monitoring of intervention delivery

All physiotherapists delivering physiotherapy interventions will have access to a comprehensive intervention manual and will be required to have undertaken trial-specific training. The training will include comprehensive guidance on the theory and practical delivery of the trial interventions. A rigorous quality control programme will also be conducted to ensure intervention fidelity. Quality assurance checks will be made by the trial team, who will observe treatment sessions and collect data on intervention delivery and number of treatment sessions attended, including details about the core and adaptable components, to facilitate monitoring and reporting.

### Outcome measures

The primary outcome is shoulder pain and function at 12 months measured using the Shoulder Pain and Disability Index (SPADI) total score,^37 38^ which was developed to measure current shoulder pain and disability in an outpatient setting. A systematic review of outcome measurement sets for shoulder pain trials showed that SPADI is the most commonly used measure to assess pain and disability.^39^ It has good psychometric properties,^40^ is used widely in the field and can be completed using a postal questionnaire.

Secondary outcomes (table 1) will include: subdomains of the SPADI, which are pain measured using the SPADI five-item pain subscale^37 38^ and function measured using the SPADI eight-item disability subscale^37 38^; health-related quality of life measured using the five-level version of the well-validated EuroQol Five-Dimensional Questionnaire (EQ-5D)score^41^; psychological factors measured using the Fear Avoidance Belief Questionnaire (physical activity five-item subscale)^42^ and Pain Self-efficacy questionnaire (short form)^43^; sleep disturbance measured using the Insomnia Severity Index^44^; patient global impression of change^45^; return to desired activities, including work, social life and sport activities; patient adherence to exercise; any serious adverse events (SAEs); health resource use (consultation with primary and secondary care, prescribed and over-the-counter medication use, additional physiotherapy or injection use and hospital admission); additional out-of-pocket expenses and work absence (number of sickness days).

### Adverse events

Foreseeable adverse events occurring as a result of the trial intervention(s) will not be recorded as part of the trial. Instead, participants will be provided with information on the potential adverse events resulting from exercise and corticosteroid injection (if applicable) as part of their treatment, including what they should do if they experience an adverse event, as would happen as part of standard NHS procedures. SAEs (defined as any unexpected medical occurrence than can result in death, is life threatening or results in hospitalisation or incapacity) are likely to be very rare and are highly unlikely to occur as a result of either the exercise or corticosteroid injection therapy delivered in this trial. However, if an SAE arises from the participant’s enrolment in the trial to their final visit for their allocated intervention, standard procedures for recording and reporting SAEs will then apply.

### Follow-up data collection

Measurements for the primary and secondary outcomes will be collected by postal or web-based questionnaires at 8 weeks, 6 months and 12 months after randomisation. Participants will be asked to complete the questionnaire and return it to the GRASP study team in a prepaid envelope or online as appropriate. For those who do not respond, at least one postal reminder will be sent. Telephone follow-up will be used to contact those who do not respond to the reminder or who have not fully completed the returned questionnaire.

### Data management

All data will be processed according to the Data Protection Act 1998 and all documents will be stored safely in confidential conditions. All trial-specific documents, except for the signed consent form and follow-up contact details, will refer to the participant with a unique study participant number/code and not by name. Participant identifiable data will be stored separately from study data and in accordance with local procedures. All trial data will be stored securely in offices only accessible by swipe card by the central coordinating team staff in Oxford and authorised personnel.

### Sample size

The target sample size is 704 randomised participants (176 in each treatment arm). This is based on 90% power and 1% two-sided statistical significance to detect a minimally clinically important difference of eight points on the SPADI total scale,^37^ assuming a baseline SD of 24.3 (chosen as representative of the patient population^46^). This difference is the equivalent of a standardised effect size of 0.33, which requires a sample size of 550 participants (Power Analysis and Sample Size 13, www.ncss.com). Allowing for a potential loss to follow-up at 12 months of 20% inflates the sample size to 688. The sample size has been further inflated to take into account the potential for a small clustering by physiotherapist effect in the progressive exercise group. We use an intracluster correlation coefficient (ICC) of 0.001, based on our experience with individually tailored physiotherapy interventions^47^ and expect each physiotherapist to treat approximately 20 participants in the progressive exercise group. This leads to an inflation factor of f=1+(m−1)*ICC=1+(20−1)*0.001=1.019 and increases the sample size to a total of 704 participants (176 per arm).

This sample size assumes no interaction effect and is powered for the two main effect comparisons: (1) progressive exercise versus best practice advice and (2) corticosteroid injection versus no injection when no interaction is present. However, this number of participants will also provide 80% power and 5% two-sided significance to detect an interaction standardised effect size of 0.35, if such an interaction effect does exist. The interaction effect will be tested before the main effect comparisons are undertaken. It should be noted that a non-significant interaction effect does not preclude a smaller interaction that this study is not powered to detect.

### Statistical analysis

The primary statistical analysis will be carried out on the basis of intention to treat. The primary outcome measure is shoulder pain and function measured using the SPADI^37 38^ total score at 12 months postrandomisation. The scale is based on 13 questions, all scored on a 0–10 numerical rating scale on which 10 is the worst score, with a five-item pain subscale and an 8-item disability subscale. The subscale items and total are summed and converted to a 0–100 scale, where a higher value denotes more pain and/or disability. There will be two main effect comparisons for this 2×2 factorial trial: (1) progressive exercise versus best practice advice and (2) subacromial corticosteroid injection versus no injection.

The analysis will be undertaken using longitudinal methods in a multivariable analysis with adjustment for the baseline SPADI score, stratification factors, important prognostic factors, clustering by physiotherapists and taking into account the multiple time-points. Statistical significance will be set at the 1% level and corresponding 99% CIs will be reported for the primary outcome. For all other outcomes, 5% significance and 95% CIs will be reported. The data distribution will be formally assessed and if evidence for departure from normality is found, non-parametric techniques will be used with no adjustment (for example the Mann-Whitney test or the Kruskal-Wallis test). The secondary outcomes will be analysed using the same methodology as for the primary outcome.

#### Missing data

A linear mixed longitudinal model will be used to analyse all available data for the primary outcome. This method can take account of missing observations either due to missed visits or to a participant leaving the study prematurely, and can also be used when the participants are not all assessed at exactly the same time-point, as the exact time for each observation is used in the analysis. Missing data will be reported and summarised by treatment arm. The distribution of missing data will be explored to assess the assumption of data being missing at random. Multiple imputations will be used, if appropriate.

### Economic evaluation

A within-trial economic evaluation will be conducted in parallel with the assessment of the clinical effectiveness of the four intervention groups. The factorial design will also allow the economic evaluation of the two primary comparisons. Health-related quality of life will be estimated using the EQ‐5D-5L.^48^ The responses to the EQ-5D will be converted into multiattribute utility scores using an approved ‘cross-walk’ to the three-level instrument and its established utility algorithm for the UK,^49 50^ or the new UK-approved five-level utility tariff, if published. The economic evaluation will be conducted from a UK NHS and Personal Social Services perspective^51^ and will compare the costs and outcomes at 12-month follow-up using the trial data. The outputs of the economic evaluation will be presented in terms of expected incremental cost-effectiveness ratios. Cost-effectiveness acceptability curves will be generated via non-parametric bootstrapping and displayed graphically, alongside cost-effectiveness planes and expected net benefit statistics. Probabilistic sensitivity analyses will be performed to explore the implications of parameter uncertainty on the incremental cost-effectiveness ratios. Subgroup analysis using predefined subgroups will investigate potential treatment moderators such as age, sex and other baseline characteristics for which cost effectiveness is predicted to be different.

The at-the-margins approach (without interactions) may treat the factorial trial as though it were two overlapping two-arm randomised trials and may effectively ignore the factorial design as it assumes that factors have purely additive effect. If there is no interaction, ignoring interactions is statistically efficient, answering two questions with the same sample size required for one. However this form of analysis gives biased or misleading results if there is any interaction. Regression analysis provides a convenient way to evaluate interactions and main effects. Including covariates within regression facilitates adjustment for baseline imbalance, which may be particularly important for factorial trials. For the purpose of the economic evaluation, we will investigate the possible interactions with quality-adjusted life-years and costs. The distribution of costs and benefits and correlation between costs and effects will also be considered. If factors are thought to have a multiplicative effect, a general linear model may be appropriate in transformed data.

## Ethics and dissemination

Ethics approval was obtained from the Berkshire B Research Ethics Committee (REC: 16/SC/0508) (22 November 2016), approved by the UK Competent Authority, the Medicines and Healthcare Regulatory Agency (MHRA) (EuDRACT: 2016-002991-28) and prospectively registered (ISRCTN: 16539266). A data monitoring and ethics committee has been appointed to independently review data on safety, protocol adherence and recruitment to the trial in accordance with the DAta MOnitoring Committees: Lessons, Ethics, Statistics (DAMOCLES) charter. Direct access to research data will be granted to authorised representatives of the Sponsor (University of Oxford), regulatory authorities or the host institution for monitoring and/or auditing of the study to ensure compliance with regulations. Summary results data will be included on the EudraCT database within 12 months of the end of the trial. General release will be 5 years after the end of the trial, to allow the investigators sufficient time to complete and report additional analyses of the data set. Trial findings will inform NHS clinical practice for the management of patients with a rotator cuff disorder and its results will be published as a monograph as part of the National Institute of Health Research Health Technology Assessment journal series and in a high-impact open-access journal, in accordance with Consolidated Standards of Reporting Trials (CONSORT) and Template for Intervention Description and Replication (TIDieR^52^) guidelines. Trial materials will be made available via the trial website on completion of the trial. Prior to formal publication, we will inform the participants of the trial results. Participants will be asked if and how they would like to be informed of the results as part of the consent process.

## Supplementary Material

Reviewer comments
