# Supplementary material for: Clinical and cost-effectiveness of progressive exercise compared with best practice advice, with or without corticosteroid injection, for the treatment of rotator cuff disorders: protocol for a 2x2 factorial randomised controlled trial (the GRASP trial)
Source: BMJ Open. 2017 Jul 17;7(7):e018004. doi: 10.1136/bmjopen-2017-018004 (PMC5683303; doi:10.1136/bmjopen-2017-018004)
Supplement: Supplementary Appendix 1 [file bmjopen-2017-018004supp001.pdf]

Appendix 1: British Elbow and Shoulder Society (BESS) Diagnostic Algorithm

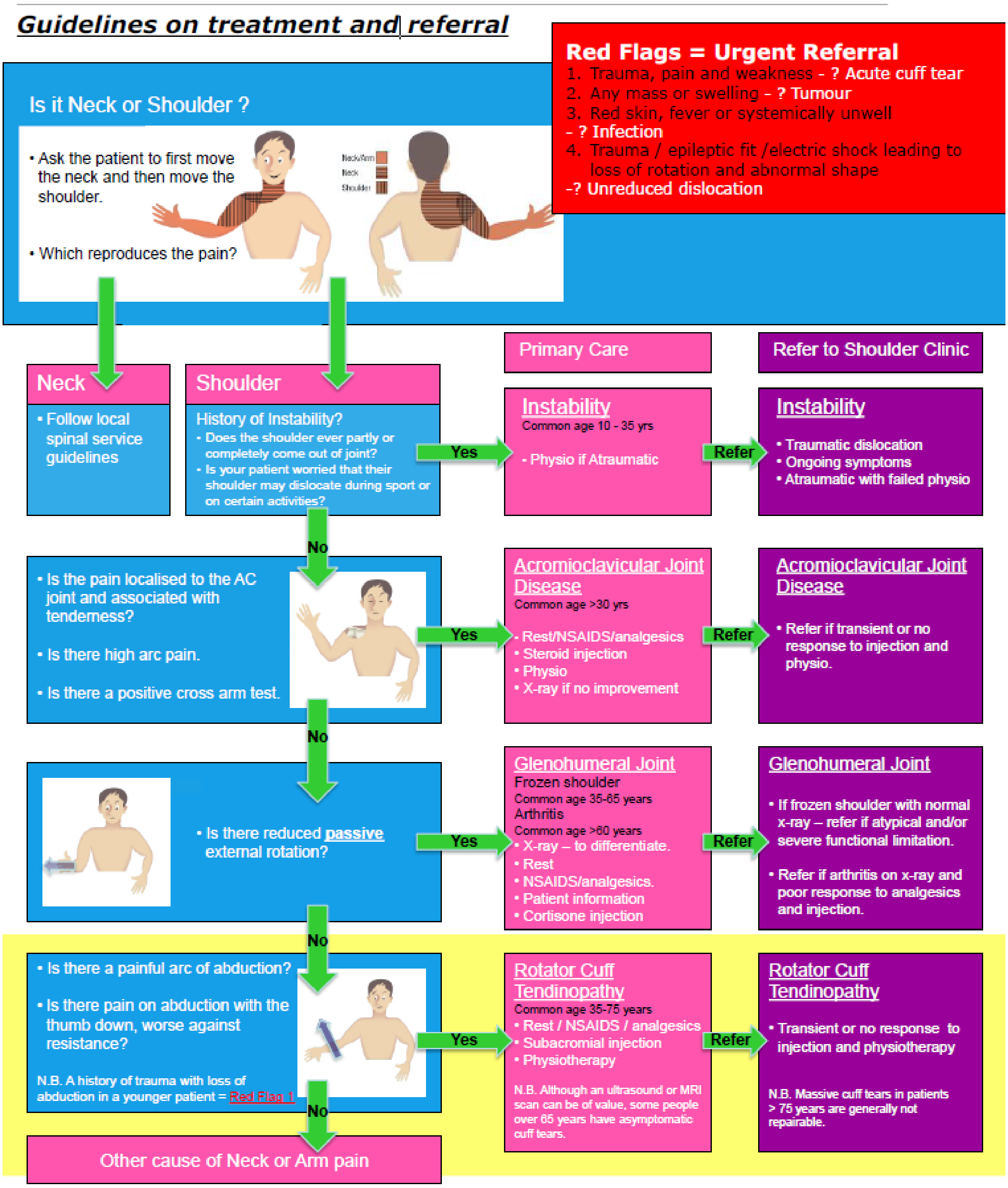

Eligibility assessment will be carried out using the diagnostic algorithm developed by the British Elbow and Shoulder Society (BESS) and other professional bodies (e.g., Royal College of Surgeons, Chartered Society of Physiotherapy, British Orthopaedic Association) and is part of the NICE-accredited standards (3) to confirm when a diagnosis of rotator cuff disorder is highly likely, based on a patient's history and simple shoulder tests (5).
