# Supplementary material for: Clinical and cost-effectiveness of progressive exercise compared with best practice advice, with or without corticosteroid injection, for the treatment of rotator cuff disorders: protocol for a 2x2 factorial randomised controlled trial (the GRASP trial)
Source: BMJ Open. 2017 Jul 17;7(7):e018004. doi: 10.1136/bmjopen-2017-018004 (PMC5683303; doi:10.1136/bmjopen-2017-018004)
Supplement: Reviewer comments [file bmjopen-2017-018004.reviewer_comments.pdf]

PEER REVIEW HISTORY

BMJ Open publishes all reviews undertaken for accepted manuscripts. Reviewers are asked to complete a checklist review form (**http://bmjopen.bmj.com/site/about/resources/checklist.pdf**) and are provided with free text boxes to elaborate on their assessment. These free text comments are reproduced below.

ARTICLE DETAILS

**TITLE (PROVISIONAL)**	Clinical and cost effectiveness of progressive exercise compared to best practice advice, with or without corticosteroid injection, for the treatment of rotator cuff disorders: protocol for a 2x2 factorial randomised controlled trial (the GRASP trial)
**AUTHORS**	Hopewell, Sally; Keene, David; Maia Schlüssel, Michael; Dritsaki, Melina; Dutton, Susan; Carr, Andrew; Hamilton, Willie; Hansen, Zara; Jaggi, Anju; Littlewood, Chris; Soutakbar, Hessam; Heine, Peter; Cureton, Lucy; Barker, Karen L.; Lamb, Sarah

This article was not externally reviewed at BMJ Open. Protocols that have been independently assessed prior to submission to BMJ Open are usually fast-tracked to publication on the grounds that further substantial changes will not be possible. This independent assessment will usually be external, independent review for both a substantial grant award from a non-commercial or government funder and ethics approval.
